# The effects of whole-body electromyostimulation (WB-EMS) in comparison to a multimodal treatment concept in patients with non-specific chronic back pain—A prospective clinical intervention study

**DOI:** 10.1371/journal.pone.0236780

**Published:** 2020-08-21

**Authors:** Karl Lorenz Konrad, Jean-Pierre Baeyens, Christof Birkenmaier, Anna Helena Ranker, Jonas Widmann, Johannes Leukert, Lisa Wenisch, Eduard Kraft, Volkmar Jansson, Bernd Wegener

**Affiliations:** 1 Department of Orthopaedic Surgery, Physical Medicine and Rehabilitation, Ludwig-Maximilians- University Munich (LMU), Munich, Germany; 2 Department of Physiotherapy, Human Physiology and Anatomy, Vrije Universiteit Brussel (VUB), Brussel, Belgium; Friedrich-Alexander-Universitat Erlangen-Nurnberg Medizinische Fakultat, GERMANY

## Abstract

**Background:**

According to present guidelines, active exercise is one key component in the comprehensive treatment of nonspecific chronic back pain (NSCBP). Whole body electromyostimulation (WB-EMS) is a safe, and time-effective training method, that may be effective in NSCBP-patients.

**Methods:**

In this prospective and controlled nonrandomized clinical study, two therapeutic approaches were compared. One group received 20 minutes WB-EMS per week. An active control group (ACG) received a multimodal therapy program. A third group included subjects without back pain. To all groups, the following measurement instruments were applied: Numeric Rating Scale (NRS), Oswestry Disability Index (ODI), North American Spine Society Instrument (NASS); SF 36 survey and measurements for muscular function and postural stability. In the EMS-group: T0: baseline; T1: at 6 weeks; T2: at 12 weeks and T3: at 24 weeks. In the ACG: T0 baseline and T1 after 4 weeks.

**Results:**

In the intervention group, 128 patients with low back pain were enrolled, 85 in the WB-EMS group and 43 in the ACG. 34 subjects were allocated to the passive control group. The average age was 58.6 years (18–86 years). In the EMS group, the NRS (1–10) improved statistically and clinically significantly by 2 points. The ODI was reduced by 19.7 points. The NASS and most of the SF 36 items improved significantly. In the multimodal treatment group, only the muscular function improved slightly.

**Conclusion:**

Our data support the hypothesis that WB-EMS is at least as effective as a multimodal treatment, which is often referred to as being the golden standard. Therefore WB-EMS may be an effective and, with 20 min./week training time, very time-efficient alternative to established multimodal treatment models.

## 1. Introduction

Non-specific chronic back pain (NSCBP) is the leading chronic disease globally and it forces more people out of their job than diabetes, heart disease, hypertension, respiratory disease, asthma, and neoplasm together [1, 2]. It is the number one cause for years lived with disability (disability-adjusted life-years) [3]. NSCBP may occur over the entire life span, but it is most common in the age between 40 and 69. Women are more often affected than males. The highest prevalence is observed in countries with high income and high human development index (30.3% vs 18.2% low income) such as the USA, Great Britain, France or Germany [4]. In about 90% of cases, no anatomical pain-correlate beyond age-normal spinal degeneration can be established, which is why it is termed non-specific [5]. Based on these facts, the prevention and rehabilitation of NSCBP-patients seem to be of crucial importance in present-day health care.

A sedentary lifestyle, which is more common in countries with a high human development index, shows a significantly increased incidence of recurring back pain [6–8]. The reduced physical activity, which characterizes the sedentary lifestyle, leads to a reduction of muscle power and strength in particular of the trunk [9].

The function of the muscles providing stability to the trunk plays an essential role in the development and maintenance of chronic back pain. Several studies have shown that there is a clear link between back pain and trunk muscle insufficiency [9–11, 2, 6]. In addition, patients suffering from chronic back pain often avoid to move, once pain is persistent. They fear to induce pain by moving and develop fear avoidance behavior (kinesiophobia). Hence these patients are caught in a vicious circle: Movement avoidance leads to loss of muscular abilities, that in turn favors pain, and pain again leads to the avoidance of physical activity [12–14].

Consequently, the key component of interventions for the treatment of these patients, is to break the vicious circle with active exercise, as has been stated in various European and national guidelines [5, 15]. Strength training programs appear to be superior to other interventions in the treatment of NSCBP [16, 17].

Patients with NSCBP often suffer from further limitations of their mobility due to osteoarthritis of the large joints. Our busy modern lifestyle seems to be the main barrier to overcome the vicious circle and start physical training [18]. Therefore, there is an urgent need to enable physically passive NSCBP-patients to exercise easier and more time-effectively.

Whole body electromyostimulation (WB-EMS) is a novel training method for strengthening the muscles, which became increasingly popular within the fitness sector [19]. With this method, multiple muscle groups are stimulated simultaneously. Beyond the proximal extremities, it is mainly the trunk muscles which are being trained. Numerous studies have already demonstrated a positive effect of WB-EMS on the performance of the targeted muscles [20–30]. The skeletal muscles exhibit a significant increase in muscle mass, power and maximal strength. An increase in oxygen intake at the anaerobic threshold, as well as a reduced blood pressure was accomplished. 20 minutes of training once a week was shown to be effective. A study on patients with heart failure (NYHA II-III) supports that even very low subjective training intensity shows a 14% increase in muscle mass [31]. It has been proven that WB-EMS training activates deep spinal stabilizing muscles, such as the lumbar multifidus and the transversal abdominal muscles [32]. There is also evidence that simultaneous stimulation of the abdominal and lumbar muscles is the most effective method to achieve activation of the deep spine-stabilizing muscles [32, 33]. Based on these facts, it appears appropriate to apply this kind of training to patients with NSCBP.

The studies by Kemmler et al. [34] and by Weissenfels et al. [35] showed a significant decrease in pain intensity and an increase in maximum trunk strength in LBP patients. But these findings were compared to a passive control group only.

The aim of this prospective controlled clinical trial was to investigate the effects of WB-EMS on Patients with NSCBP over the course of time of 6 months and compare the observed effects to an established multimodal treatment. In addition, the differences to a group without back pain was determined.

## 2. Materials and methods

### 2.1. Study approval and registration

The individual in this manuscript has given written informed consent to publish these case details. This study was carried out at the Department of Orthopedics, Physical Medicine and Rehabilitation of the Ludwig-Maximilians-University Munich (LMU), Germany, during the period between 04/2017 and 12/2018. This clinical trial was registered in the German clinical trials register (ID: DRKS00011896) and is therefore listed in the International clinical trials registry of the World Health Organization. The study was also approved by the University Ethics Committee (Project number 547–16). The study complies with the ethical guidelines of the Helsinki Declaration (last modification 2013).

### 2.2. Research-questions

The study goals were specified by formulating the following three research-questions and the according hypotheses:

**Question 1**: Is there a difference in the Numeric rating scale (NRS)between subjects with NSCBP and Subjects with a “healthy back”?

H0: Healthy subjects do not differ from patients NSCBP in terms of the parameters studied
H1: Healthy subjects differ from patients with NSCBP in terms of the parameters studied

**Question 2:** Do the symptoms and functional parameters in NSCBP-Patients improve through the WB-EMS intervention?

H0: The therapy with WB-EMS training does not improve the symptoms and functional parameters of NSCBP-Patients in terms of the parameters studied.
H1: The therapy with WB-EMS training improvs the symptoms and functional parameters of NSCBP-Patients in terms of the parameters studied.

**Question 3:** Do the results on outcome variables differ between WB-EMS and an established multimodal low back pain program?

H0: Results of WB-EMS do not differ to established multimodal back pain concept.
H1: Results of WB-EMS do not differ to established multimodal back pain concept.

### 2.3. Participants

Participants were recruited through posters and flyers in the outpatient clinics of the university hospital, and by an article in the in-house journal. The pre study estimation of minimal sample size of n = 103 (69 in EMS, 34 in control group) via PS Power and Sample Size Calculations 3.0 was therefore exceeded.

All participants were informed about the study before they gave their informed consent to participate. The study complies with the ethical guidelines of the Helsinki Declaration (last modification 2013). The recorded data was encrypted and is stored according to the German Data Protection Act, at the Department of Orthopaedic Surgery, Physical Medicine and Rehabilitation at Ludwig-Maximilians-University Munich (LMU), Munich, Germany. All participants were instructed to not change their routine physical activity, beside changes due to the study.

#### 2.3.1 Inclusion / exclusion criteria

Inclusion criteria for all participants:

- only occasional sports / spine gymnastics / physiotherapy
- no psychiatric disorders
- no neuromuscular diseases
- no diagnosed balance problems / vertigo
- no neurologically detectable coordination disorders
- no known psychiatric diagnoses

Inclusion criteria for NSCBP participants:

- diagnosed with non-specific chronic low back pain by a physician
- low back pain for more than 3 months

Inclusion criteria for participants without low back pain:

- no back pain within the preceding 3 months
- no back pain with structural correlates in imaging for the previous 3 years

In addition, for all participants specific, absolute contraindications were defined: Acute diseases, bacterial infections or inflammatory processes, recent operations (preceding 2 months), vascular stents or bypasses in situ for less than 6 months, untreated high blood pressure, pregnancy, electrical implants (e.g. pacemaker), known malignancies (last 5 years), abdominal wall or inguinal hernias.

If one of the following specific, relative contraindications was present, the attendees were only allowed to participate, if the responsible physician gave a statement of safety: Acute back problems without diagnosis, acute neuralgia, herniated discs, implants (prostheses) not older than 6 months, diseases of the internal organs (especially kidneys), cardiovascular diseases, epilepsy, open skin injuries, wounds, eczema, burns, medication intake.

### 2.4. WB-EMS training program

In this whole-body training method, belts and vests are used, which contain several electrodes for the application of the electric current on all major muscle groups. The WB-EMS device used for this study (miha bodytec GmbH, Gersthofen, Germany) has a total electrode surface area of 2800 cm^2^ and stimulates about 16 muscle groups on the proximal extremities and on the trunk simultaneously. In a systematic review, Filipovic et al. were able to identify proven electric parameters for effective training regimens [21]. Based on these findings, the following electrical parameters were used: Four seconds pulse duration and four seconds pulse break (50% duty cycle), impulse with 350 microseconds, impulse frequency of 85 Hertz with a symmetric bipolar, rectangular pulse form.

#### 2.4.1 Training schedule

The training program was compiled according to the guidelines of Kemmler et al. [36]. The initial session consisted of five minutes impulse familiarization followed by 12 minutes of reduced training. The perceived intensity of electric stimulation was categorized using a scale from “0 ≙ nothing at all” to “10 ≙ extremely strong (maximum)” [37, 38]. During the first six sessions, the limit for the perceived intensity was set to at maximum “5 ≙ strong”. From the sixth to the ninth session, perceived intensity was gradually increased to levels between “7 ≙ very strong” and in maximum “8”. Because the tolerance for the electric current is increasing, the current was adjusted every time, the subject reported a decrease of perceived intensity. The duration of all sessions, except the first, has been 20 minutes once a week. The complete training period lasted six months.

**Fig 1:**
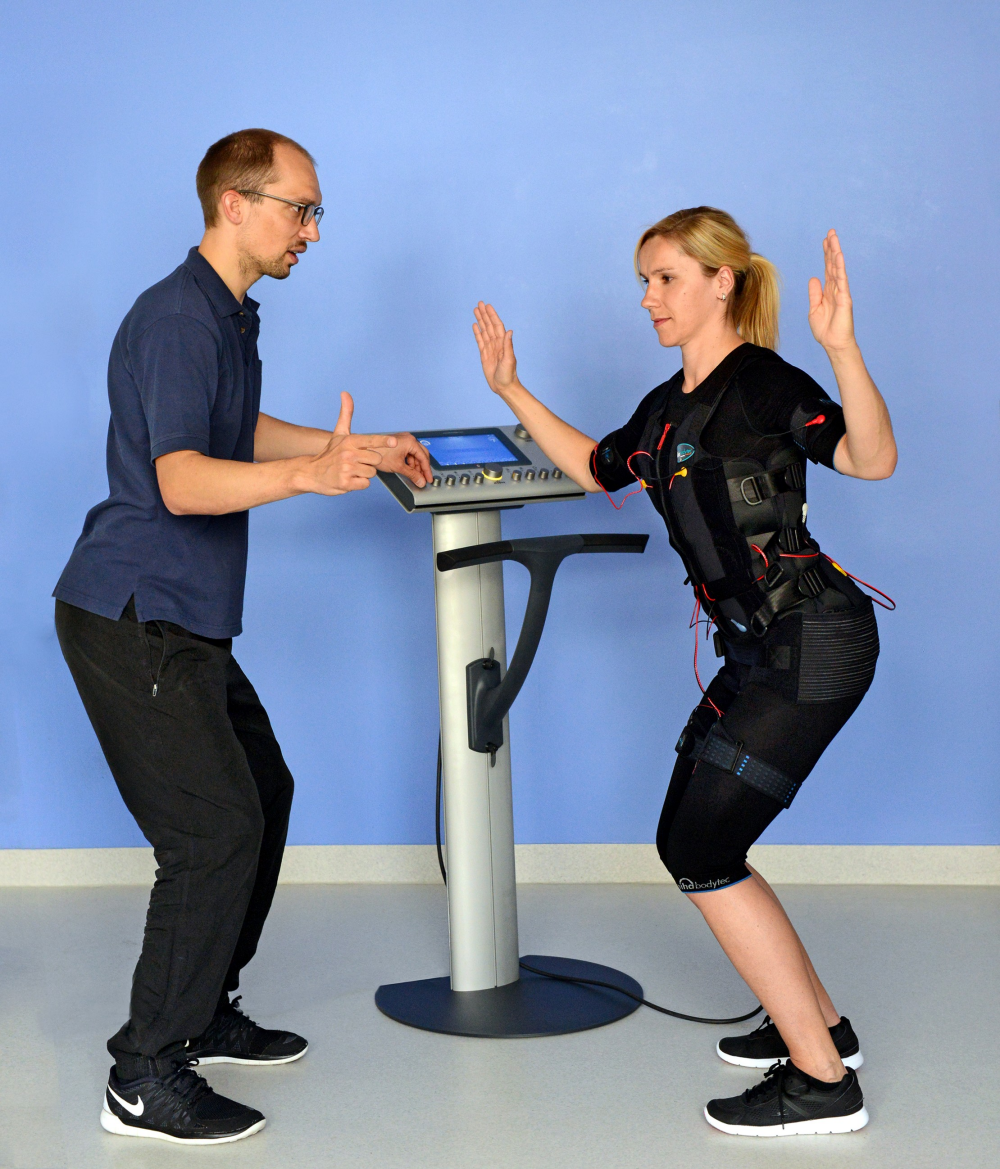
Training with WB-EMS. Typical therapy setting, reproduced by employees of the institute. The subject is wearing wests and belts to apply the electric stimulation and is supervised by a trained physiotherapist.

Dynamic EMS-Training had shown to be more effective than static EMS-training [39]. Therefore, using the “back strengthening” program of the EMS device, the subjects performed the following dynamic exercises, while the pulse duration (4sec.): Squat, butterfly reverse left, butterfly reverse right, trunk rotation left, trunk rotation right, left diagonal crunches, right diagonal crunches, straight crunches, trunk extension and table position. All exercises were carried out in an upright position, without external load (see Fig 1). Every training session was guided by a WB-EMS certified physiotherapist. In addition, the video-guidance of the device with animated avatars was used. Normally one Therapist guided 2 Subjects (in maximum 3 Subjects) at the same time.

#### 2.4.2 Assessments

The assessments were applied at the following times: T0 (baseline): before treatment, T1: after 6 weeks of treatment, T2: after 12 weeks of treatment and T3 after 6 months of treatment (complete training period).

On each of these assessments pain and additional parameters were determined with the following, well established questionnaires: Numeric rating scale (NRS), Short Form 36 Health Survey (SF 36), North American Spine Society Instrument (NASS), Oswestry Disability Index (ODI) and Hospital Anxiety and Depression Scale (HADS). Postural stability and functional (muscular) performance were assessed by means of Leonardo Mechanography^®^ and the MFT-S1-Checklist^®^ gyroscope.

### 2.5. ACG: Multimodal low back pain program

All subjects in the active control group were included in an established multimodal therapy program for the treatment of NSCBP-Patients, in an outpatient clinics concept. The program was composed according to the recommendations of the national German and the European guidelines [15, 5], and consisted of physiotherapy, physical therapy, psychotherapy, occupational therapy and education. Patients stayed at the clinics from Monday through Friday from 9 am. to 4 pm. and received coordinated treatments. The treatment period in total was 4 weeks.

The following objectives were pursued in the various interventions:

Physiotherapy:

The patients were instructed in targeted strength training (segmental stabilization), in order to improve the body stabilizing muscle corset. Coordination and balance, posture and body awareness were the aims of these trainings. Endurance training and hydrotherapy with relaxing and strengthening exercises were also applied.

Psychology:

Psychotherapists trained the patients in self-helping strategies and relaxation techniques.

Occupational Therapy:

The attendees were educated in ergonomic behavior for the activities of daily live.

Physical therapy:

Patients tried different simple methods from the areas of hydrotherapy and thermotherapy to check individually whether one of these techniques could relief or mitigate their subjective symptoms, in order to then implement these techniques in everyday life.

Medical information and education:

In order to provide a more self-determined therapy regime, the subjects received lectures with detailed information about NSCBP, as well as about drug and non-drug therapies. Specific problems were addressed in one-to-one discussions and examinations with a physician. The exact composition of the multimodal program was published in the article of Letzel, Angst and Weigl (see Table 1 there) [40], just the target treatment region was adjusted for NSCBP.

**Table 1:** Mean values of NSCBP-patients (at T0) and the subjects with “healthy back”.

Test No	Healthy subjects		NSCBP patients		Difference
	Mean	+/-	Mean	+/-	
**NRS**	**0.55**	0.68	**4.48**	2.16	-3.93
**ODI_Score**	**2.69**	4.42	**33.96**	16.22	-31.27
**SF-36 social role functioning. (0–100)**	**95.97**	8.77	**75.3**	23.6	20.67
**SF-36 bodily pain (0–100)**	**88.45**	13.21	**46.48**	17.83	41.97
**SC: av. P rel (W/kg)**	**4.65**	0.63	**3.1**	0.84	1.55
**CRT: t per repet. (sec)**	**1.56**	0.39	**2.15**	0.91	-0.59
**SF-36 vitality (0–100)**	**74.03**	15.94	**49.28**	17.17	24.75
**TRT: V max (m/s)**	**0.6**	0.18	**0.51**	0.23	0.09
**TRT: F tot max rel (N/Kg)**	**13.39**	1.93	**12.92**	2.85	0.47
**TRT: P tot max rel (W/kg)**	**0.68**	0.25	**0.54**	0.25	0.13
**NASS-Lumbar_Neurological**	**1.21**	0.39	**2.09**	1.08	-0.88
**NASS-Lumbar_Pain**	**1.27**	0.31	**2.52**	0.8	-1.25
**HADS Depression positiv**	**0**	0	**0.18**	0.39	-0.18
**SF-36 physical functioning (0–100)**	**96.21**	6.09	**65.38**	22.39	30.83
**SF-36 physical role functioning (0–100)**	**99.19**	4.49	**53.72**	42.46	45.47
**SF-36 general health perception (0–100)**	**80.71**	14.1	**57.09**	16.94	23.62
**SF-36 mental health (0–100)**	**82.45**	10	**68.92**	16.98	13.53

#### 2.5.1. Assessments

In the ACG group, assessments were performed at T0 (Baseline): before treatment and T1: after four weeks of treatment (completion of the intervention period). During each of these assessments, pain and condition were determined using the same tests as in the WB-EMS group, but the HADS Score was assessed only at T0.

### 2.6 Peer group: Subjects with “healthy back”

In order to determine current reference values, the negative control group (no back pain) was examined with the same assessment procedure as the WB-EMS-group.

### 2.7. Primary outcome variables

Since pain is the main symptom in NSCBP, the NRS was set as the primary outcome variable. The NRS is widely used as an outcome measure for subjective pain intensity. Subjects rate their pain on a scale from 0 (no pain) to 10 (worst imaginable pain). The NRS has been shown to have sufficient psychometric strengths and predictive validity as a measure of pain intensity, to be used in chronic pain research [41].

### 2.8 Secondary outcome variables

#### 2.8.1 Mechanography

The mechanography measurement has proven to be a save and objective method to assess muscle function while performing functional movements [42]. Variations of the ground reaction force over the course of time is recorded during a specific movement. Outcome Parameters like body mass, jumping height, maximum forces, velocity and power output are calculated from the recorded data. In the present study, one of the common devices, the Leonardo Mechanograph ground reaction force Plate (GRFP) and the Leonardo Stair, were used (Leonardo GRFP STD, Leonardo Stair A, Novotec Medical GmbH, Pforzheim, Germany). Data acquisition and Data analysis were realized with the Leonardo Mechanography Software, Version 4.4, research edition (Novotec Medical GmbH, Pforzheim, Germany).

#### 2.8.2 Single two leg jump

The Single two leg jump (S2LJ) test is a measurement of ground reaction force (GRF) while performing counter movement jump. This method is known as a safe and reliable test of the musculoskeletal function of the lower limb [43–45, 42, 46]. To perform the S2LJ, participants were instructed to perform a counter movement jump. According to the manual of the device, participants were asked to jump once as high as possible, using both legs and to land on the forefoot. Arm swing was allowed. From the recorded Data Pmax. _rel_. and EFI were calculated using the corresponding software.

#### 2.8.3 Chair-rising test

The Chair-rising test (CRT) is a measurement of the ground reaction forces while performing a complete sit to stand and stand to sit cycle [42]. The average time needed to perform one rise and sit cycle (t per repet.) is the main outcome of the test. For the CRT the subjects were sitting on the standardized bench with 45 cm of height (GRFP equipment), with the arms crossed before the chest. Subjects were instructed to get up completely three times immediately after another, as fast as possible and sit down again right away. They had to keep the arms crossed during this procedure.

#### 2.8.4 Trunk rise test

With the Trunk rise test (TRT) the ground reaction force is recorded, while the upper body is lowered to about 90° and raised again, both ways as fast as possible. This test is part of the Leonardo Software (research edition), and it is described as a test of the musculoskeletal function of the lower trunk. From a standing starting position, with crossed arms, the subjects were instructed to bend forward about 90 degrees and rise again, as fast as possible. The maximum speed (V max), the maximum force per kg bodyweight (F tot max _rel_) and the maximum power per kg bodyweight (P tot max _rel_) were calculated.

#### 2.8.5 Stair climb test

In the stair climb test (SC) with the “Stair A”, the ground reaction force is assessed, while a 5 step stair ascend [42]. The participants were instructed to ascend the stair as fast as possible by using every step. They were not allowed to use the handrail. The relative maximum force (Fmax rel./g), the average relative maximum power and the relative (overall) average power (av. P _rel_) as well as the average horizontal velocity (av. v hor) was calculated.

#### 2.8.6 Postural stability (S1-Checklist)

To assess postural stability, the MFT-S1-Checklist system (trend sport trading GmbH, Großhöflein, Austria) was used. The S1 Checklist is a monoaxial balance platform with an integrated angle sensor. It has a standardized instability with a tilt angle of 12° to both sides. For measurements the platform was used in longitudinal as well as in transverse position, to access balance and postural capacity in frontal (left-right) and in sagittal (front-back) plane. The S3 device is an established and validated system with the outcome variables stability index (STI) and sensorimotor index (SMI) [47]. The STI describes postural stability on an instable platform considering body symmetry and sensorimotor regulatory capacity on a scale from 1 (very good) to 9 (very poor). The SMI is a scale for the number and amplitude of compensation movements from 1 (very good = few and small compensation movements) to 9 (very poor = many and large movements).

#### 2.8.7 Clinical questionnaires

The following standardized and well-established questioners were used:

- Oswestry Disability Index (ODI) [48]
- North American Spine Society Lumbar Spine Outcome Assessment Instrument (NASS) [49].
- Short Form 36 Health Survey (SF36) [50]
- Hospital Anxiety and Depression Scale (HADS) [51]

### 2.9. Data analysis

Data analysis was performed using the SPSS 22 software package (IBM Corp., Armonk, USA). Since the main outcome variables such as NRS, ODI, SF36 and most other outcome variables are non-parametric scaled [52], all calculations were carried out with non-parametric statistics in order to present the data consistently. For the comparison of two measurements the Mann-Whitney-U-Test, and for more than two measurements the Kruskal-Wallis-test was performed. Pearson correlation coefficient (*r*) was calculated from Z scores of these tests to display the effect size (Formula: $r=|\frac{Z}{\surd n}|$). For comparison with Cohen’s *d* (Standardized mean difference [SMD]) in other publications, we converted *r* into *d* by using the formula $d=\frac{2r}{\sqrt{1}-r^{2}}$ [53]. The level of significance was set at p < 0.05 (5%). The smallest analyzed unit was 1 subject (corresponding to the unit at inclusion). Missing data was replaced with the mean value of its class in the time row, to not influence the mean values. The statistical analysis was blinded. The study worker who carried out the calculations therefore had no information, if data was assigned to test or to control group.

## 3. Results

### 3.1. Sample description and flow of participants

206 individuals had been screened for eligibility, 184 found to be eligible and 162 individuals declared their participation in the study. Of the recruited individuals, 128 NSCBP patients and 34 healthy subjects met the inclusion criteria and were included in the study (Total n = 162). Initially, 85 subjects (62 female, 23 male) with a mean age of 55.7 (±13.7) years, were participating in the WB-EMS group and completed the T0 assessment. 80 (94%) subjects completed T1, 77 (91%) participants completed T2 and 62 (73%) subjects finished the whole 6-month period and completed T3 also. Two drop-outs from T0 to T1 and one drop-out in the T1-T2 period appeared as the participant became pregnant. The main reasons for drop-out, were the long duration of the study and changes of participants daily tasks during the study-period, with a resulting time conflict, followed by unexpected worsening of a co-existing disease, pregnancy and at least by health problems caused by an accident.

Out of 43 (31 female, 12 male) participants with a mean age of 63.5 (±11.0) years on T0, completed 41 (95%) subjects T1 of the multimodal ACG. There were no statistically significant differences at baseline between the WB-EMS group and the ACG in terms of pain intensity (NRS) and ODI.

**Fig 2:**
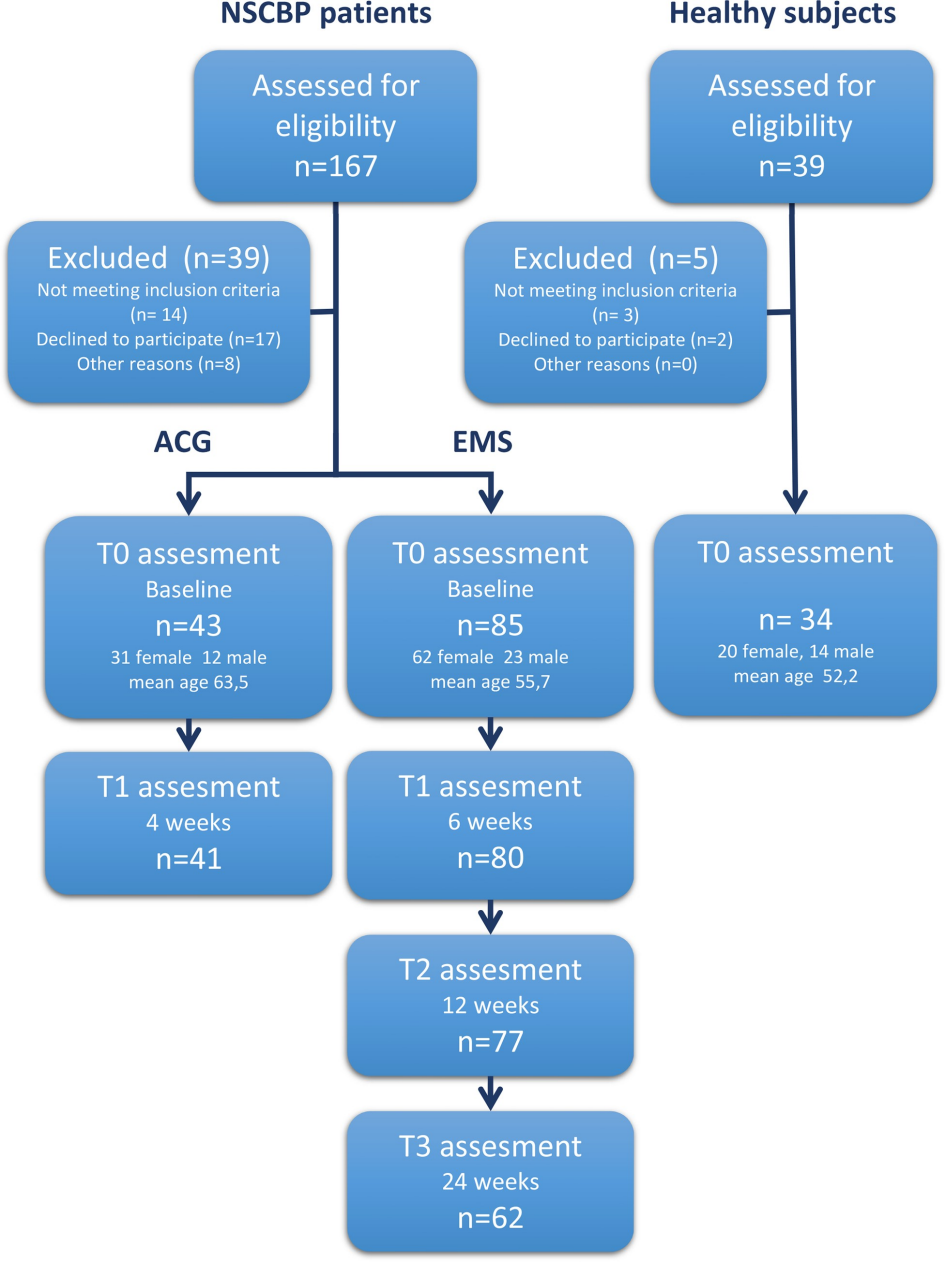
The flow of participants throughout the study. NSCBP = nonspecific chronic back pain; n = number of subjects in subgroup; EMS = WB-EMS-Group; ACG = active control group.

34 subjects (20 female, 14 male) with a mean age of 52.2 (±12.1) years and a healthy back participated in the PCG (See Fig 2). Between the WB-EMS group and the PCG, there were no statistically significant differences in terms of age. During the active period of this study, no adverse or unintended effects were observed. The collected Data contained some missing values, but the extend of missing values was always below 2% (1,48%-1,81%) in ACG and WB-EMS group, and below 1% (0,98%) in PCG.

### 3.2. Results concerning 1st question

When comparing the subjects with NSCBP and the subjects with a “healthy back” at T0 using the Mann-Whitney U-test, we observed significant differences in the primary outcome (NRS) and most of the secondary parameters (see Table 1).

Therefore, the alternative hypothesis “there is a difference” was accepted for these outcome parameters.

For the secondary parameters with no significant differences between NSCBP patients and healthy subjects, the null-hypothesis of no difference was accepted (see Table 1). These parameters were not analyzed further.

### 3.3. Results concerning 2nd question

The effectivity of the WB-EMS and ACG interventions over the course of time was evaluated using the Kruskal-Wallis-test for multiple comparisons between the measurement points. Significant improvements in the primary outcome parameter (NRS) and in 14 secondary parameters were identified in the WB-EMS group. The values with the corresponding delta are shown in Table 2.

**Table 2:** Mean values and changes of the WB-EMS group.

Test No.	T0 = baseline		T1 = 6 weeks				T2 = 12 weeks				T3 = 24 weeks			
	Mean	+/-	Mean	+/-	Delta	ES	Mean	+/-	Delta	ES	Mean	+/-	Delta	ES
**NRS**	**4.45**	2.02	3.07**	1.96	-1.38	0.3	2.87**	2.02	-1.58	0.4	2.40**	1.75	-2.04	0.5
**ODI_Score**	**33.8**	16.58	17.72**	12.23	-16.08	0.5	18.01**	13.37	-15.8	0.5	14.10**	10.25	-19.7	0.6
**SF-36 social role functioning. (0–100)**	**76.13**	23.41	**84.09**	18.87	7.96	0.2	84.20*	20.66	8.07	0.2	92.67*	14.63	16.54	0.4
**SF-36 bodily pain (0–100)**	**48.89**	17	57.92*	19.52	9.03	0.4	60.86**	20.24	11.97	0.3	66.22**	18.36	17.33	0.4
**SC: av. P rel (W/kg)**	**3.28**	0.81	**3.47**	0.78	0.2	0.1	3.57*	0.82	0.29	0.2	3.85*	0.77	0.58	0.4
**CRT: t per repet. (sec)**	**1.87**	0.55	**1.71**	0.43	-0.16	0.2	1.55*	0.42	-0.32	0.4	1.54*	0.33	-0.33	0.4
**SF-36 vitality (0–100)**	**49.86**	16.46	54.93*	20.84	5.07	0.2	**54.93**	19.92	5.07	0.1	62.41*	17.3	12.55	0.3
**TRT: V max (m/s)**	**0.51**	0.22	**0.55**	0.22	0.04	0.2	0.59*	0.22	0.08	0.2	**0.57**	0.19	0.05	0.2
**TRT: F tot max rel (N/Kg)**	**13.55**	3.18	**13.82**	1.76	0.27	0.2	14.10*	1.9	0.55	0.2	**13.94**	1.81	0.4	0.2
**TRT: P tot max rel (W/kg)**	**0.58**	0.26	**0.64**	0.24	0.07	0.2	**0.68**	0.45	0.11	0.2	**0.64**	0.22	0.06	0.2
**NASS-Lumbar_Neurological**	**2.04**	1.02	**1.82**	0.94	-0.21	0.1	1.67*	0.87	-0.37	0.2	1.57*	0.81	-0.47	0.2
**NASS-Lumbar_Pain**	**2.48**	0.78	2.14*	0.73	-0.34	0.2	2.16*	0.79	-0.33	0.2	1.94**	0.7	-0.54	0.3
**HADS Depression positiv**	**0.14**	0.35	**0.14**	0.35	0	na	**0.18**	0.39	0.04	na	**0.11**	0.32	-0.03	0.1
**SF-36 physical functioning (0–100)**	**68.86**	21.79	75.51*	21.53	6.65	0.2	75.88*	21.19	7.03	0.2	78.87*	17.94	10.01	0.2
**SF-36 physical role functioning (0–100)**	**58.33**	41.7	**67.43**	38.73	9.1	0.1	76.76*	33.36	18.43	0.2	79.55*	33.02	21.21	0.2
**SF-36 general health perception (0–100)**	**58.2**	16.57	**62.89**	17.74	4.69	0.1	**62.21**	17.87	4.02	0.1	67.42*	16.49	9.22	0.2
**SF-36 mental health (0–100)**	**70.89**	15.58	**72.88**	16.23	1.99	0.1	**73.94**	16.31	3.05	0.1	78.28*	13.53	7.38	0.3

*Significant changes from T0 (p<0.05)

**Significant changes from T0 (p<0.01)

**Fig 3:**
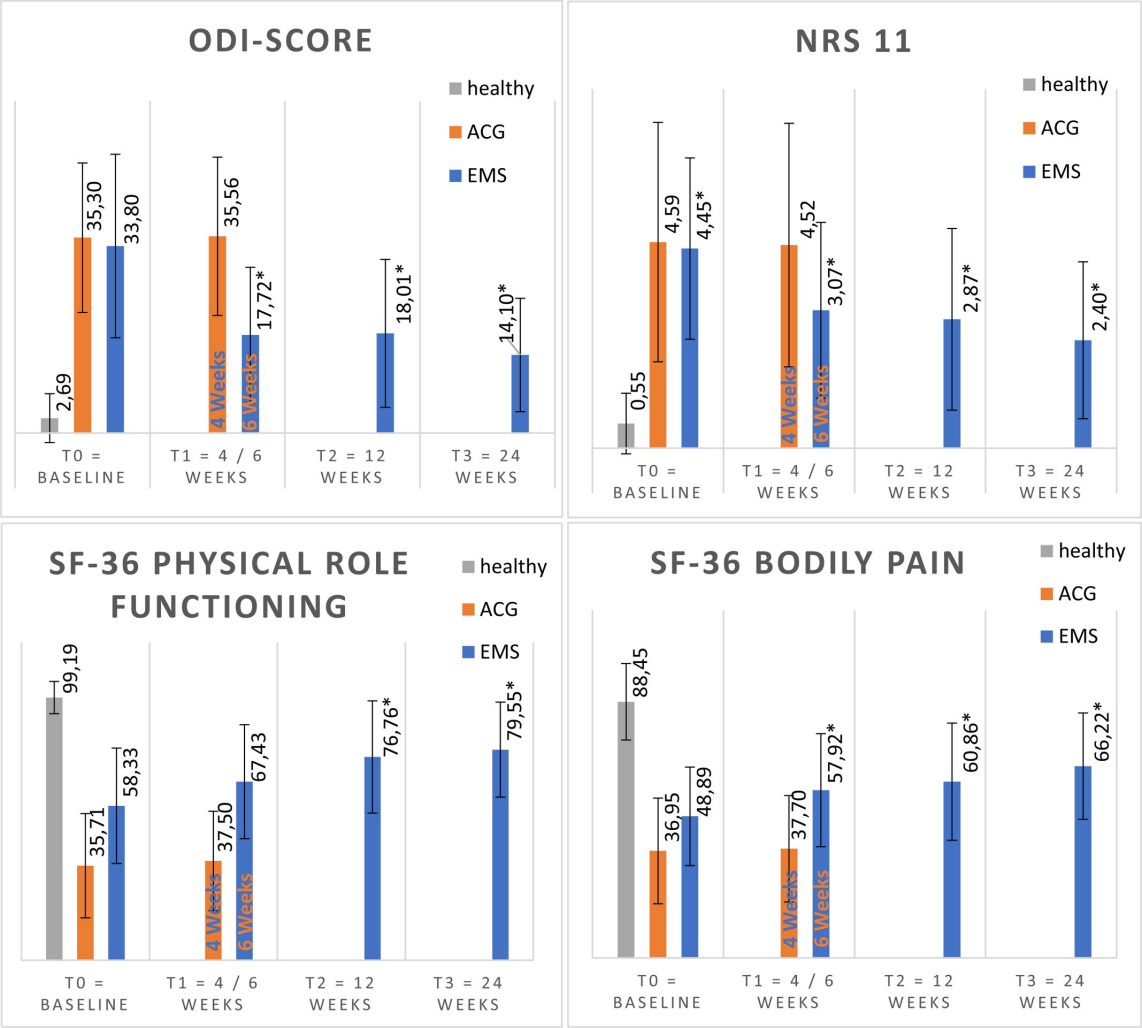
The 4 variables with the highest relative improvement in WB-EMS group. Mean values (columns) and standard deviation (black lines) of outcome scales Oswestry Disability Index (ODI), Numeric Rating Scale (NRS) and the subscales of the short form 36 (SF36). ACG = active control group. EMS = WB-EMS group, healthy = subjects without back pain.

The improvements with the highest significant relative changes (in percentage from baseline) in the WB-EMS group were observed in the following outcome variables: ODI-score (-58%, *r* = 0.6). NRS (-46%, *r =* 0.5). SF-36 physical role functioning (36% *r =* 0.35). SF-36 bodily pain (35%, *r =* 0.4) and SF-36 vitality (25%, *r =* 0.3) (see Fig 3).

The alternative hypothesis “WB-EMS improves low back pain” was accepted for all outcome parameters which showed significant changes

**Fig 4:**
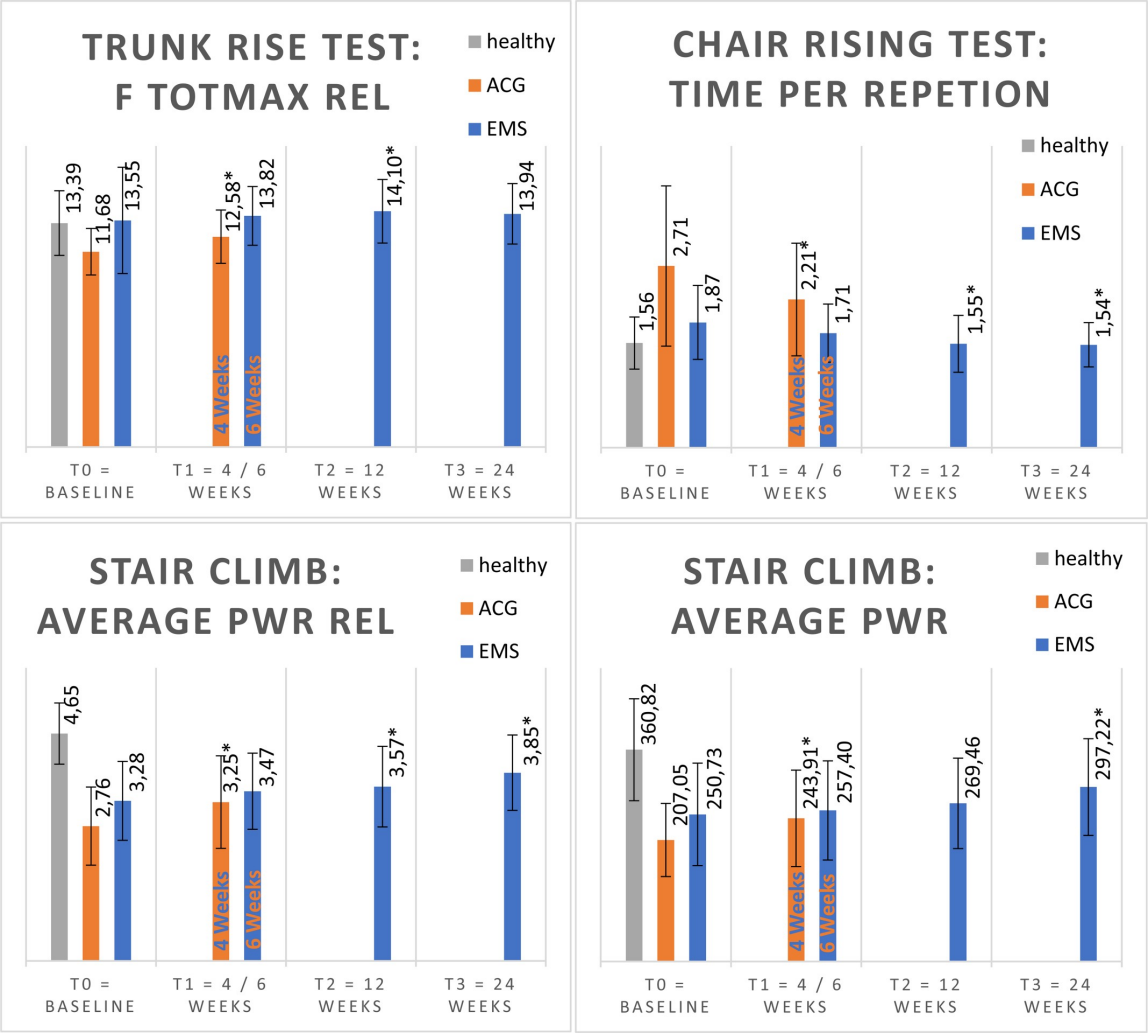
The 4 variables with the highest relative improvement in ACG. Mean values (columns) and standard deviation (black lines) of outcome scales Trunk rise test: relative total maximal force (F totmax rel) in N/kg, Chair Rising Test: time per repletion in seconds, Stair Climb Test: average relative power (average Pwr rel) in W/kg and average power (average Pwr). ACG = active control group. EMS = WB-EMS group, healthy = subjects without back pain.

In the ACG group, the primary outcome (NRS) did not change significantly, only four of 22 secondary outcome parameters changed significantly over the observed period. (see Fig 4 and Table 3).

**Table 3:** Mean values and changes of the ACG group.

Test No.	T0 = baseline		T1 = 4 weeks			
	Mean	+/-	Mean	+/-	Delta	ES
**NRS**	**4.59**	2.67	**4.52**	2.71	-0.07	0.0
**ODI_Score**	**35.3**	13.52	**35.56**	14.31	0.26	0.0
**SF-36 social role functioning. (0–100)**	**72.02**	24.66	**71.25**	25.03	-0.77	0.0
**SF-36 bodily pain (0–100)**	**36.95**	18.24	**37.7**	18.38	0.75	0.0
**SC: av. P rel (W/kg)**	**2.76**	0.8	3.25*	0.95	0.49	0.3
**CRT: t per repet. (sec)**	**2.71**	1.2	2.21*	0.84	-0.5	0.3
**SF-36 vitality (0–100)**	**46.98**	20.03	**45.33**	19.02	-1.65	0.0
**TRT: V max (m/s)**	**0.51**	0.24	**0.48**	0.18	-0.02	0.0
**TRT: F tot max rel (N/Kg)**	**11.68**	1.39	12.58*	1.59	0.9	0.3
**TRT: P tot max rel (W/kg)**	**0.47**	0.2	**0.53**	0.2	0.06	0.2
**NASS-Lumbar_Neurological**	**2.63**	1.51	**2.57**	1.62	-0.05	0.0
**HADS Depression positiv**	**0.32**	0.48	**0.33**	0.48	0.02	0.0
**SF-36 physical functioning (0–100)**	**51.64**	19.68	**53.22**	18.77	1.58	0.0
**SF-36 physical role functioning (0–100)**	**35.71**	41.51	**37.5**	41.75	1.79	0.0
**SF-36 general health perception (0–100)**	**52.76**	18.08	**53.4**	18.3	0.64	0.0
**SF-36 mental health (0–100)**	**61.14**	20.24	**59.8**	19.79	-1.34	0.0
**SC: av. P (W)**	**207.05**	62.33	243.91*	82.2	36.86	0.3

*Significant changes from T0 (p<0.05).

## 3.5. Results concerning 3rd question

There was a considerable difference in the number of variables that improved in the intervention groups. In the WB-EMS group, the primary outcome parameter (NRS) and 14 secondary outcome parameters improved significantly. In contrast, in the ACG, only four secondary outcome parameters showed significant improvement but not the primary outcome (NRS).

To explore the difference in the effectiveness of intervention in the groups WB-EMS and ACG, the relative delta of these significant change between the measurements was compared. These changes all occurred at almost the identical relative amount (see Table 4).

**Table 4:** Absolute and relative delta in parameters which showed significant changes in the ACG in comparison to the values in WB-EMS group.

	ACG		WB-EMS	
	Delta T0-T1	Delta rel.	Delta T0-T3	Delta rel.
**CRT: t per repet. (sec)**	0.5s	18%	0.3s	18%
**SC: av. P rel (W/Kg)**	0.5 W/kg	18%	0.6 W/kg	18%
**SC: av. P (W)**	37Watt	18%	47Watt	19%
**TRT: F tot max rel (N7Kg**	0.9N/kg	8%	0.4 N/kg	3%

## 4. Discussion

The aim of this study was to investigate the effectiveness of WB-EMS on patients with NSCBP over the time course of 6 months, and to compare the effects with the outcome of an active control group (ACG) which completed an established multimodal treatment program. The findings of this study show a significant improvement of the WB-EMS group in primary outcome parameters, especially in scales of back pain associated questionnaires. The reduction of the degree of disability by nearly 20% in the ODI score was very impressive and clinically relevant [48]. The effect of these changes was strong with a standardized mean difference (SMD) of 1.3 (*r* = 0.6). The NRS back pain values improved with an SMD of 1.2 (*r* = 0.5) and by 2 points, an improvement which is clinically relevant [54]. Also the musculoskeletal tests as secondary outcome variables, such as Trunk-Rise-Test, Chair-Rising-Test and the Stair-Climb-Test test showed improvements, which may indicate an improvement of the muscular strength and function in the lower extremities and trunk [55, 56].

When the current research project was initiated in August 2016, a literature search on PubMed yielded no results for literature of WB-EMS on back pain. In the meantime, one clinical trial and one retrospective analysis have targeted this topic [34, 35]. Although, the study of Kemmler et al. [34] is an analysis of their previously conducted WB-EMS trials, which originally had not focused on NSCBP, the authors also observed significant reductions in pain intensity. The SMD of 0.8 in that study was slightly lower than in the present study (NRS SMD = 1.2 (*r* = 0.5)). These improvements were confirmed in the only identified clinical trial aiming on WB-EMS and chronic back pain. Weissenfels et al. showed a significant decrease in pain intensity and an increase in maximum trunk strength in n = 15 LBP patients (age between 40 and 70 years) compared to a passive control group [35]. The SMD of the observed improvement for pain intensity was 0.75. The mean pain level at baseline of this study was 2.75 points on NRS in the WB-EMS group, whereas in the current study the mean pain level was 4.45 points on NRS. The question arises, whether there are differences in the effect of WB-EMS on NSCBP, depending on the initial pain intensity.

Weissenfels et al. [35] mentioned in their report also two nonpublished master theses, which may additionally support the finding of pain reduction, even though the inclusion criteria’s were not clearly limited to participants with nonspecific back pain [57, 58]. In this context, the present study provides additional support to the concept of pain reduction in NSCBP patients by means of WB-EMS.

We were not able to identify any other clinical trial, that compares WB-EMS with a multimodal ACG. The ACG employed in the present study represents an established intervention method, that conforms to the current evidence-based guidelines [5, 15]. The multimodal low back pain concept is characterized by interventions of different disciplines in a high density and is widely seen as the reference for assessing the success in the therapy of NSCBP. However, the improvements achieved in the ACG reached a level of statistical significance only in 4 of 22 outcome variables. Especially the changes in the pain scores of the questionnaires did not reach significance. Opposed to this finding, the improvements in the WB-EMS group reached statistical significance in 15 outcomes, especially in NRS and in all other pain-related scores.

The Single two leg jump (S2LJ) testing is known as reliable test of musculoskeletal function of the lower limb [43–45, 42, 46]. A strong relationship between dynamic and isometric strength of the lower extremities and jumping performance has been shown [59, 60]. EMS training is known to strengthen preferably the fast twitch fibers, and therefore to improve preferably explosive strength [22, 61]. In the current WB-EMS protocol the lower limbs were also trained with that method and as a result an improvement in jumping performance was expected. However, these results only showed tendencies and did not reach statistical significance.

A relationship between muscular function, back pain and sit-to-stand performance has previously been recognized [62, 63]. Runge et al. have also shown, that an average time of more than 2.5 seconds in CR test correlates with an enhanced risk of falling [64]. Also, in this study the average time improved in both intervention groups. This may indicate an improvement of the relevant muscular system.

The SC test has shown to be a reliable test [65, 66], with clinical relevance for leg power impairments [55]. SC represents an activity of daily living and therefore, an improvement in this test may have a high Impact of the everyday life of back pain patients. Both groups were benefiting from the training and improved their SC performance.

Some studies show a reduced body balance in back pain subjects compared to healthy individuals [67]. In the current study, it was not possible to distinguish between back pain participants and subjects with a “healthy back” using the MFT-S1-Checklist system. These results are consistent with the recent study by our own group [68].

The TRT was designed to test the musculoskeletal function of the trunk, but until now there was no published evidence for this test. In the current study, a correlation between the TRT and the outcomes of the questionnaires was observed. The strongest correlation was observed between TRT Ptot max rel and SF36 bodily pain (Spearman's rho: 0,307). Further and more detailed investigation is needed to provide stronger evidence for this testing method.

HADS-Score was assessed only on T0 because a period of 4 weeks was considered to be too short for an intervention-related effect on that scale, this was also suggested by previous examinations on the same multimodal program, in the same laboratory (unpublished doctoral thesis). However, the HADS values at T0 should enable a better baseline comparison of the examined groups.

As the two observed treatment methods are very different in scheduling and density, the intension was to compare outcome parameters pre and post treatment, regardless of the treatment duration. Because of the long duration of WB-EMS intervention, we planned more assessments to provide a better insight in the longitudinal view. For a comparison between the groups, only the changes between T0 and last measurement should be considered.There are some limitations to our study: 1.) Due to the considerable differences in the organization of the two intervention-programs, it was impossible to randomize the subjects and therefore, participation-bias might have been introduced. In contrast, the relation between female and male participants, is very likely not a bias since it corresponds well to the epidemiological distribution of NSCBP [17]. 2.) The two programs differed significant in therapy density. The one program had a very high density of presence at the institute (5 whole days a week, 4 weeks) and the other one a very low density (30 minutes/week). Therefore, more subjects were able to implement the low-density method (WB-EMS) into their daily life. It was difficult to find subjects who had the opportunity to stay away from work for 4 weeks in the ACG. For that reason, it was not reached the same number of subjects in the ACG as in the EMS group. But both groups exceeded the pre estimated necessary sample size. 3.) In our setting it was not possible to develop a blinding mechanism for the participants of two so different intervention concepts, therefore there could have been uncompensated nonspecific effects. 4.) Only single center design. 5.) An objective reassessment of the multimodal ACG after 6 months was not possible, because a considerable number of participants have been already involved in further Therapy programs. 6.) Various programs for the therapy of NSCBP patients have been developed. In our case, we compared a multimodal concept, which is very expensive with an inexpensive and time saving method. The effect in terms of pain and quality of life should be analyzed over a period of several years, to allow a final statement. A comparison with other concepts would be also necessary.

## 5. Conclusion

This study demonstrates significant and clinically relevant improvements in the symptoms of NSCBP by use of a dedicated WB-EMS training program. Parameters of musculoskeletal function improved similarly to the ACG. Our data support the hypothesis that WB-EMS is at least as effective as the multimodal low back pain concept used in this trial. Therefore WB-EMS may be an effective and, with a training time of 20 minutes per week, a very time-efficient alternative to traditional multimodal therapies.

## Supporting information

S1 Checklist TREND statement checklist. (PDF)

S1 File Initial sample size estimation. (PDF)

S2 File Anonymized data file. (CSV)

S1 Study protocol (DOCX)
