# Supplementary material for: The effects of whole-body electromyostimulation (WB-EMS) in comparison to a multimodal treatment concept in patients with non-specific chronic back pain—A prospective clinical intervention study
Source: PLoS One. 2020 Aug 21;15(8):e0236780. doi: 10.1371/journal.pone.0236780 (PMC7446884; doi:10.1371/journal.pone.0236780)
Supplement: S1 File — (PDF) [file pone.0236780.s002.pdf]

Dr. Michael Lauseker
IBE
Machioninistr. 15
81377 München
Telefon: 089 / 44007-4496
Telefax: 089 / 44007-7491
lauseker@ibe.med.uni-muenchen.de

München, 17.08.2016

Berechnung der Fallzahl

Für die Studie werden 2 Variablen als gleichberechtigte primäre Zielgrößen verwendet: Die Differenz in der Spunghöhe (in cm) beim Single 2 Leg Jump Test sowie die Differenz bei der benötigten Kraft (in kN) beim Trunk Rise Test zwischen der Messung zu Zeitpunkt t0 und Zeitpunkt t2. Beide Zielgrößen werden als normalverteilt angesehen.

Es wurde ein globales Signifikanzniveau von 5% gewählt, die Korrektur für die 2 Zielgrößen erfolgte mittels Bonferroni-Adjustierung, es wird also für jeden Test ein Signifikanzniveau von 2,5% verwendet. Für diese Studie wurde eine Power von 80% veranschlagt. Es wird von einem Verhältnis von 2:1 zwischen Experiment- und Kontrollgruppenpatienten ausgegangen. Alle Vergleiche sollen als zweiseitige t-Tests durchgeführt werden.

Als sekundäre Testgrößen werden die maximale Kraft (in kN) und die Leistung (in W/kg) beim Single 2 Leg Jump Test sowie die Aufrichtzeit (in s) und die Leistung (in W/kg) beim Trunk Rise Test betrachtet. Diese werden nicht in die Fallzahlplanung einbezogen, da sie lediglich explorativ betrachtet werden sollen.

Da es sich um eine bislang schlecht untersuchte Patientenkohorte handelt, besteht erhebliche Unsicherheit bezüglich der geschätzten Standardabweichungen. Daher wird nach 20 Patienten eine verblindete Sample Size Re-Estimation, wie von Friede und Kieser (2006) beschrieben, im „unrestricted design“ durchgeführt.

Die nachfolgende Tabelle gibt einen Überblick über die Annahmen, die der Fallzahlplanung zugrunde liegen, sowie die benötigte Fallzahl nach aktuellem Stand.

	Zu zeigender Unterschied	Erwartete Standardabweichung	Benötigte Fallzahl in der Experiment bzw. Kontrollgruppe
Spunghöhe beim Single 2 Leg Jump Test	5 cm	6,4 cm	48 bzw. 24
Kraft beim Trunk Rise Test	0,2 kN	0,29 kN	62 bzw. 31

Für die Auswertung werden also nach derzeitigem Stand insgesamt 93 Patienten benötigt, davon 62 aus der Experimentgruppe. Da pro Patient zwei Messungen benötigt werden, sollte eine zusätzliche Reserve von 10% rekrutiert werden um drop-outs aufzufangen. Damit erreichen wir eine Fallzahl von 103 Patienten.

Die Fallzahlplanung erfolgte mit *PS Power and Sample Size Calculations 3.0*.

Literatur:

Tim Friede & Meinhard Kieser - Sample Size Recalculation in Internal Pilot Study Designs: A Review. *Biometrical Journal 48 (2006) 4, 537–555.*

Dr. Michael Lauseker
