# Supplementary material for: The effects of whole-body electromyostimulation (WB-EMS) in comparison to a multimodal treatment concept in patients with non-specific chronic back pain—A prospective clinical intervention study
Source: PLoS One. 2020 Aug 21;15(8):e0236780. doi: 10.1371/journal.pone.0236780 (PMC7446884; doi:10.1371/journal.pone.0236780)
Supplement: S1 Study protocol — (DOCX) [file pone.0236780.s004.docx]

**Study protocol**

The following protocol is the Initial Version witch was submitted to and approved by the ethics committee of the LMU Munich, and had been published prior the conduct of the study at the German study register file (DRKS).

Changes from the initial Protocol are marked in green (added text) and red (~~removed text)~~ in this document, and was reported to the ethics-commission as well.

# 1. Protocol in original Language (German)

Please see English translation in next chapter.

## Titel der Studie

Effekte elektrischer Ganzkörper Muskelstimulation auf Menschen mit chronisch unspezifischen Rückenschmerzen. (EMBUR)

## Allgemeinverständliche Kurzbeschreibung

1. Einleitung:
Von einigen Firmen wird mit der Wirksamkeit einer Ganzkörper -Muskelstimulation für Fitnesstraining geworben. Eine starke Muskulatur soll vor verschiedenen Krankheitsbildern des muskel- und Skelettsystems schützen. Das gilt als erwiesen. Wir möchten in unserer Untersuchung Patienten mit chronischem Rückenschmerz dahingehend untersuchen, ob dieses Training auch für sie einen positiven Effekt hat.

2. Eingangs- und Abschlussuntersuchung:
Zur Beurteilung der Stabilität bzw. der Fähigkeit der Koordination der Muskulatur stehen verschiedene medizinische Verfahren zur Verfügung. Die von uns verwendeten Geräte wurden genau dafür entwickelt und sind dafür zugelassen. Es gibt jedoch bislang keine ausreichenden wissenschaftlichen Untersuchungen, ob die vorgeschlagene Trainingsmethode mit den geplanten Messungen neben dem Trainingserfolg auch zu den angestrebten medizinischen Effekten führt. Sie werden bei den Fitnesstests und beim nachfolgenden Training stets von uns angeleitet. Zusätzlich erheben wir im Rahmen eines Fragebogens, der später anonymisiert ausgewertet wird, Daten über die Leistungsfähigkeit Ihrer Muskulatur und Ihre gesundheitlichen Beschwerden. Die Untersuchung wird durchgeführt an einer Gruppe von Patienten mit chronischen Rückenschmerzen und einer gesunden Kontrollgruppe.

3. Training:
Sie werden durch ausgebildete Anleiter bei Ihrem Training begleitet. Es entstehen Ihnen durch die Studie keine Kosten. Das Training findet einmal wöchentlich als Ergänzung zu Ihrem sonstigen Programm statt. Dazu erhalten Sie einen mit Elektroden zur Muskelstimulation besetzten Anzug und eine mit dem Trainingsgerät verbundene Weste. Unter Anleitung werden dann körperliche Übungen durchgeführt. Die Muskulatur wird durch die Stromimpulse, die Sie als Kribbeln wahrnehmen, zu einer verstärkten Kontraktion stimuliert. Bitte halten Sie sich streng an die Anleitung, um gesundheitliche Risiken zu vermeiden. Das Training findet über 6 Monate statt.

4. Risiko-/Nutzenabwägung:
Training mit elektrischer Muskelstimulation ist im Leistungssport und in der Medizin seit Jahren etabliert. Daher weiß man sehr gut, dass negative gesundheitliche Effekte bei korrekter Anwendung nicht befürchten sind. Sofern sich die erwarteten positiven Effekte auf Ihre körperliche Leistungsfähigkeit und Ihre Gesundheit ergeben, steht Ihnen zukünftig ein Trainingsverfahren zur Verfügung, welches Sie selbständig im Wissen um dessen Effekte nutzen können.

5. Widerrufsrecht:
Sie haben die Möglichkeit, jederzeit ohne Angaben von Gründen und ohne Nachteile befürchten zu müssen, die Teilnahme zu beenden.

## Wissenschaftliche Kurzbeschreibung

In der klinischen ~~prospektiv randomisierten~~prospektiven kontrollierten Studie sollen zwei verschiedene Therapiekonzepte hinsichtlich ihrer Effektivität auf chronischen unspezifischen Rückenschmerz gegenüber einer Kontrollgruppe verglichen werden. Die erste Gruppe erhält eine „klassische“ Therapie mit krankengymnastischer Übungsbehandlung unter selbständiger Durchführung von isometrischen und isotonischen Übungen zum Training der Muskulatur unter fachkundiger Anleitung. Die zweite Gruppe erhält eine Trainingstherapie unter personalisierter Anleitung mit assistiver elektrischer Muskelstimulation (EMS). Eine Kontrollgruppe wird ohne spezifische Intervention mit identischem Untersuchungsprotokoll aus „rückengesunden“ Probanden gebildet.
Da die beiden Gruppen organisatorisch und Inhaltlich so stark voneinander abweichen war eine Verblindung auch auf Grund von Problemen mit der Synchronisation mit der LWS-Tagesklinik nicht möglich.

- (federführende) Ethikkommissions Vorlage-Nr.:  547-16, Ethik-Kommission der Medizinischen Fakultät der Ludwig-Maximilians-Universität München

# Untersuchte Krankheit/Gesundheitsproblem

- ICD10:   M54.9 -  Rückenschmerzen, nicht näher bezeichnet
- ICD10:   M54.8 -  Sonstige Rückenschmerzen

# Interventionsgruppen/Beobachtungsgruppen

- Arm 1:   „klassische“ Therapie mit krankengymnastischer Übungsbehandlung unter selbständiger Durchführung von isometrischen und isotonischen Übungen zum Training der Muskulatur.
- Arm 2:   EMS- Trainingstherapie unter personalisierter Anleitung mit assistiver elektrischer Muskelstimulation (WB-EMS)
- Arm 3:   Gesunde Kontrollgruppe wird ohne spezifische Intervention mit identischem Untersuchungsprotokoll aus „rückengesunden“ Probanden gebildet.

# Charakteristika

- Studientyp:  Interventionell
- Studiendesign Zuteilung:  Kontrollierte, nicht randomisierte Studie
- Verblindung:  Offen
- Kontrolle:  Aktive Kontrolle (wirksame Behandlung der Kontrollgruppe), Kontrollgruppe erhält keine Therapie
- Studienzweck:  Therapie
- Gruppenzuteilung:  ~~Parallelverteilung~~Andere

## Primärer Endpunkt

Remission der chronisch unspezifischen Rückenschmerzen während oder unmittelbar nach der Intervention.


T0: vor Beginn
T1: 6 Wochen nach Beginn
T2: 12 Wochen nach Beginn
T3: 24 Wochen nach Beginn

jeweils:

Biomechanische Tests:
MFT S3-Check
Leonardo Stair
Leonardo GRFP: Trunk Rise, multi 2Leg Hop, CRT

Fragebögen:
NASS( North American Spine society), VAS (Visuelle
Analogskala), Oswestry Disability Index, SF 36 -Fragebogen, HADS (Hospital Anxiety and Depression Scale, schmerzfokussierte deutsche Fassung)

## Sekundärer Endpunkt

Verbesserung der Biomechanischen Parameter während oder unmittelbar nach der Intervention.


T0: vor Beginn
T1: 6 Wochen nach Beginn
T2: 12 Wochen nach Beginn
T3: 24 Wochen nach Beginn

jeweils:

Biomechanische Tests:
MFT S3-Check
Leonardo Stair
Leonardo GRFP: Trunk Rise, multi 2Leg Hop, CRT

# Länder in denen Studienteilnehmer rekrutiert werden

- DE:   Deutschland

# Rekrutierungsstandorte

- Universitätsklinikum  LMU München,  München

# Rekrutierung

# Geplante Studienteilnehmeranzahl gesamt:  ~~90~~140

- Monozentrisch/Multizentrisch:  Monozentrisch
- National/International:  National

## Einschlusskriterien

- Geschlecht:  Beide, männlich und weiblich
- Mindestalter:  18   Jahre
- Höchstalter:  kein Höchstalter

## Weitere Einschlusskriterien

Chronische Rückenpatienten:
- Low back pain score nach NASS und Oswestry
- Kriterien für chronischen Rückenschmerz (Schmerzen > 3 Monate)
- sporadisch Sport/ Wirbelsäulengymnastik/ KG durchführen

## Ausschlusskriterien

- psychiatrischen Erkrankungen
- Beeinträchtigung der Muskelfunktion, muskuläre Erkrankungen
- diagnostizierte Gleichgewichtsstörungen/Schwindel
- neurologisch nachweisbaren Koordinationsstörungen

# 2. English translation of study protocol

Effects of electrical whole body muscle stimulation on people with chronic nonspecific back pain (EMBUR)

Brief Summary in Lay Language

1 Introduction:
Some companies are promoting the effectiveness of whole body muscle stimulation for fitness training. A strong musculature is supposed to protect against various pathologies of the musculoskeletal and skeletal system. This is proven. We would like to examine in our study, whether this training has a positive effect on patients with chronic back pain as well.

2. Inception and final examination:
Various medical procedures are available to assess the stability or the ability to coordinate the muscles. The devices we use have been developed and approved for this purpose. However, there are yet insufficient scientific investigations on whether or not the proposed training method, with the planned measurements, leads to the desired medical effects, in addition to the training success. During the fitness tests and the following training, you will be always guided by us. In addition, we collect data on the performance of your muscles and your health problems, within the scope of a questionnaire, which will be analyzed anonymously. The study is performed on a group of patients with chronic back pain and a healthy control group.

3. Training:
You will always be guided by trained instructors, during your training. The study does not incur any costs. The training takes place once a week as a supplement to your other program. For this purpose, you will receive a suit with electrodes for muscle stimulation and a vest connected to the training device. Under guidance, physical exercises will be performed. The muscles will be stimulated by the electrical impulses, which you perceive as tingling, to an increased contraction. Please follow strictly the instructions, to avoid any health risks. The training takes place over 6 months.

4. Risk / benefit assessment:
Training with electrical muscle stimulation has been established for years in sports and medicine. Therefore, it is well known and negative health effects are not expected while correct application. If the expected positive effects on your physical performance and your health will result, a new training procedure will be available to you, which you can use independently in the knowledge of its proven effects.

5. Right of revocation:
You can terminate the participation at any time, without reasons and without disadvantages

Brief Summary in Scientific Language

In this clinical prospective ~~randomized~~controlled study, two different therapeutic approaches are compared, on patients with chronic non-specific low back pain, in terms of their effectiveness. The first group receives a "classic" treatment of active physiotherapy with exercise treatment under independent implementation of isometric and isotonic exercises for muscle training, under expert guidance. The second group will receive a training therapy, under personalized instruction, with assistive electrical muscle stimulation (WB-EMS). A control group is formed without any specific intervention on identical test protocol from subjects with a “healthy back”.
Since the two groups differ so much in terms of organization and content, blinding was not possible due to problems with the synchronization with the LWS day clinic.

Health Condition or Problem studied

- ICD10:   M54.9 -  Dorsalgia, unspecified
- ICD10:   M54.8 -  Other dorsalgia

Interventions/Observational Groups

- Arm 1:   "classic" treatment of active physiotherapy with exercise treatment under independent implementation of isometric and isotonic exercises for muscle training
- Arm 2:   EMS training therapy, under personalized instruction, with assistive electrical muscle stimulation (WB-EMS)
- Arm 3:   control group which is formed without any specific intervention on identical test protocol from subjects with a “healthy back”.

Characteristics

- Study Type:  Interventional
- Allocation:  ~~Randomized~~Non-randomized controlled trial
- Blinding:  Open (masking not used)
- Control:  Active control (effective treament of control group), Control group receives no treatment
- Purpose:  Treatment

Primary Outcome

Remission of chronic nonspecific back pain during or immediately after the intervention.


T0: before beginning
T1: 6 weeks after beginning
T2: 12 weeks after beginning
T3: 24 weeks after the start

each:

Biomechanical tests:
MFT S3 check
Leonardo Stair
Leonardo GRFP: Trunk Rise, multi 2Leg Hop, CRT

Questionnaires:
NASS (North American Spine society), VAS (Visual
Analogue Scale), Oswestry Disability Index, SF 36 survey, HADS (Hospital Anxiety and Depression Scale, pain-focused German version)

Secondary Outcome

Improvement of the biomechanical parameters during or immediately after the intervention.


T0: before beginning
T1: 6 weeks after beginning
T2: 12 weeks after beginning
T3: 24 weeks after the start

each:

Biomechanical tests:
MFT S3-Check
Leonardo Stair
Leonardo GRFP: Trunk Rise, multi 2Leg Hop, CRT

Countries of Recruitment

- DE:   Germany

Locations of Recruitment

- University Medical Center  LMU München,  München

Recruitment

- Target Sample Size:  ~~90~~140
- Monocenter/Multicenter trial:  Monocenter trial
- National/International:  National

Inclusion Criteria

- Gender:  Both, male and female
- Minimum Age:  18   Years
- Maximum Age:  no maximum age

Additional Inclusion Criteria

Chronic Back Pain Patients:
- Low back pain score after NASS and Oswestry
- Criteria for chronic back pain (pain> 3 months)
- sporadic sports / spine gymnastics / PT

Exclusion Criteria

- psychiatric disorders
- Impairment of muscle function, muscular diseases
- diagnosed equilibrium disorders / dizziness
- neurologically detectable coordination disorders
